# The Performance of a Rapid Coronavirus Disease 2019 Antigen Test in Rural Gabon

**DOI:** 10.4269/ajtmh.24-0230

**Published:** 2025-02-18

**Authors:** Saskia Dede Davi, Dearie Glory Okwu, Lillian Rene Endamne, Teite Rebecca Hildebrandt, Rella Zoleko-Manego, Ghyslain Mombo-Ngoma, Maradona Daouda Agbanrin, Rafiou Adamou, Ayola Akim Adegnika, Selidji Todagbe Agnandji, Michael Ramharter, Johannes Mischlinger

**Affiliations:** ^1^ Center of Tropical Medicine, Bernhard-Nocht Institute for Tropical Medicine & I. Dept. of Medicine University Medical Center Hamburg-Eppendorf, Hamburg, Germany;; ^2^ German Center for Infection Research, Partner Site Hamburg-Lübeck-Borstel-Riems, Hamburg, Germany;; ^3^ Centre de Recherches Médicales de Lambaréné, Lambaréné, Gabon;; ^4^ Department of Implementation Research, Bernhard-Nocht Institute for Tropical Medicine, Hamburg, Germany;; ^5^ Institut für Tropenmedizin, Eberhard-Karls-Universität Tübingen, Tübingen, Germany

## Abstract

Access to severe acute respiratory syndrome coronavirus 2 (SARS-CoV-2) testing is limited in rural sub-Saharan Africa. We evaluated the performance of the Siemens CLINITEST^®^ rapid coronavirus disease 2019 antigen test under real-life conditions during the pandemic in rural Gabon. From August 2021 to February 2022, 277 participants were tested in Sindara and Lambaréné, Gabon, via outpatient mobile services. Of these participants, 54.6% were female, with a median age of 29 years (interquartile range: 12–55). The test performance was analyzed for the main population and for SARS-CoV-2 infected sub-populations at both study sites (Lambaréné and Sindara). We further evaluated subpopulations with higher viral loads using a cycle threshold (Ct) value restricted to <35, 30, 25, and 20. Overall test sensitivity in the main population was 33.3% (95% CI: 23.6–44.3%), improving to 83.3% (95% CI: 35.9–99.6%) with a Ct value of <20. Specificity across all populations was 100% (190/190; 95% CI: 98.1–100%). The prevalence of polymerase chain reaction-defined SARS-CoV-2 was 31.4%. The positive predictive value was 100% (95% CI: 88.1–100%), and negative predictive value was 76.6% (95% CI: 70.8–81.7%). Sensitivity in Sindara was 71.4% (95% CI: 29–96.3%) and 30% (95% CI: 20.3–41.3%) in Lambaréné. The Siemens CLINITEST^®^ demonstrated high specificity but low sensitivity overall. However, it exceeded the WHO-defined quality criteria of 80% in participants with high viral loads, making it a useful tool in resource-limited settings.

## INTRODUCTION

Severe acute respiratory syndrome coronavirus 2 (SARS-CoV-2) is an RNA virus transmitted by animals or humans. Severe acute respiratory syndrome coronavirus 2 first emerged in December 2019 in Wuhan, China, and caused coronavirus disease 2019 (COVID-19).^1^ On January 11, 2020, the WHO declared the outbreak of COVID-19 a public health emergency of international concern. On March 11, 2020, the WHO declared the COVID-19 outbreak a pandemic.^2^ As of January 10, 2024, 773,819,865 confirmed COVID-19 cases and 6,966,045 deaths have been reported globally.^3^ Additionally, as of January 10, 2024, 12,307,804 COVID-19 cases and 257,161 deaths (case fatality rate: 2.1%)^4^^,^^5^ have been reported in 55 African countries,^5^ which account for only 1.6% of confirmed global cases.^3^^,^^6^

The WHO recommends quantitative real-time polymerase chain reaction (qPCR), which detects viral RNA through nucleic acid amplification, as the diagnostic gold standard.^7^ Nucleic acid amplification tests (NAATs) confirm SARS-CoV-2 infection in suspected cases and are used for contact tracing. In sub-Saharan Africa (SSA), the performance of NAATs is typically restricted to central reference laboratories, which are generally located in cities.^8^ Thus, access to SARS-CoV-2 testing is limited, particularly for rural and resource-limited settings. Moreover, molecular testing strategies are challenging in rural settings due to the long distances from the peripheral locations to urban reference laboratories, resulting in a diagnostic turnaround time of several days. The WHO has called for research on point-of-care (POC) diagnostics in response to this challenge. Consequently, various rapid diagnostic tests (RDTs) have been developed and made commercially available.^6^^,^^7^ Therefore, infection control may be less efficient due to prolonged periods of isolation for suspected cases, delayed contact tracing, and the further spread of the infection within the country.^8^

The advantages of RDTs include their user-friendliness, cost-effectiveness, ease of use, and fast turnaround time at the point of need.^8^ This ensures the effective control of local outbreaks and helps prevent the onward transmission of SARS-CoV-2 to larger communities.

The European Commission has listed all RDTs for SARS-CoV-2 that have received regulatory approval (Conformité Européene mark) and for which their clinical performance was evaluated.^9^ One of these RDTs is the Siemens CLINITEST^®^ rapid COVID-19 antigen test (CLINITEST^®^; Siemens Healthineers AG, Erlangen, Germany), which is commercially available. The CLINITEST^®^ is a lateral flow immunochromatographic assay that detects the nucleocapsid protein of SARS-CoV-2.^10^ The CLINITEST^®^ was validated in several settings across the Northern Hemisphere.^10^^–^^13^ The reported average values for sensitivity varied considerably, ranging from 54.9% to 98.3%, which may be explained by different testing time points (at the beginning of the infection or later), the sampling of nasopharyngeal swabs, and different cycle threshold (Ct) value cut-offs (in all studies, Ct ≤30).^13^

At the same time, specificity was consistently high, ranging from 95% to 100%.^11^ Although SARS-CoV-2 RDTs serve as an alternative to qPCR, particularly in SSA, a limitation is that diagnostic test validation research has mostly been conducted in high- and middle-income countries.^8^ Only a few studies have evaluated the diagnostic performance of RDTs under field conditions in SSA. These studies reported sensitivity ranging from 45% to 88% and specificity of ≥98%.^14^^–^^16^ The diagnostic performance of RDTs in African settings may differ from that in other regions because of variations in climatic conditions, the distinct epidemiological contexts of SARS-CoV-2, and the concurrent presence of other relevant pathogens.^14^^,^^17^ We evaluated the CLINITEST^®^ in real-life conditions during a high transmission period in rural Gabon.

## MATERIALS AND METHODS

### Study design and study site.

A cross-sectional diagnostic evaluation study was conducted from August 2021 to February 2022 at two sites in Gabon to compare the diagnostic test performance of the CLINITEST^®^ under field conditions against the gold standard of qPCR. One site was the Centre de Recherches Médicales de Lambaréné (CERMEL), a national reference center for SARS-CoV-2 diagnostics with a sophisticated laboratory located in Lambaréné, Gabon. Lambaréné is a rural town in the Moyen-Ogooué Province.^18^ The other site was the Institut de Santé de Sindara (ISSA), a sentinel site of CERMEL located in Sindara, a remote village in the tropical rainforest of Gabon, in the Province of Ngounié. This village has limited access to electricity, tap water, and health facilities. It is 90 km from Lambaréné and ∼29 km from Fougamou, where the closest national test centers are located. Since October 2021, Gabon has been considered a high-risk area for SARS-CoV-2 infection. In Gabon, a curfew was imposed for the unvaccinated at night, and gatherings of more than 30 people were prohibited. Wearing masks in public was mandatory, and a negative PCR result was required to travel between districts.^19^

### Study population.

During this high transmission period, individuals at least four years of age who attended the outpatient mobile test services of ISSA or CERMEL were invited to participate in this study. No sample size calculation was performed for this exploratory study. Before any study procedures, written informed consent was obtained from all adults (age ≥18 years); caregivers obtained permission and written assent for minors. For illiterate participants, the consent form was read aloud in the presence of an impartial witness, allowing time and opportunity for further clarification and documentation by signing the informed consent. Both the consenting process and the questionnaire-based interview were conducted in French. For those who could not communicate in French, fieldworkers translated into the local language. The data obtained were entered into the electronic data bank using REDCap (REDCap Consortium, Nashville, TN) within 24 hours by trained data clerks. Subsequently, a second review of the paper-based source document was conducted. When all data were clear and conformed to good clinical practice, the investigator compared the paper-based data to the data in the electronic database and made corrections as necessary.

All participants were informed that if the qPCR test result was positive, the data would be automatically transferred to the Gabonese authorities to facilitate contact tracing and isolation in accordance with the Gabonese Ministry of Health guidelines.

### Sample collection and assessment of SARS-CoV-2.

Trained medical staff collected two nasopharyngeal swabs from all eligible participants. One nasopharyngeal swab was taken according to the CLINITEST^®^ instructions: “The swab was inserted 2–4 cm deep into both nostrils and twisted five times. Next, the swab was placed in a test tube with 10 drops of buffer solution. After 1 minute, the swab was removed, and four drops of the sample solution were applied to the test cassette. The result was available after 15 minutes.”^12^ The second swab was a nylon swab (eNat Copan Diagnostics, Brescia, Italy) containing 1 mL of liquid Amies transport medium, which was used for the qPCR assessment of SARS-CoV-2. Standard operating procedures for specimen collection (nasopharyngeal swabs), including the use of personal protective equipment, were established, and the field team was trained accordingly. Precautions were taken to ensure that the specimen was not contaminated during collection, transportation, and storage.

The RDTs were stored at temperatures below 30°C, and testing was conducted on the spot at temperatures ranging from 25°C to 30°C. Samples for the qPCR assessment were stored in the dark at temperatures between –20°C and 8°C and transported to the laboratory within a maximum of 3 days.

### Diagnostic testing by RDT and qPCR.

Rapid diagnostic testing was performed according to the manufacturer’s instructions. The study team reviewed the analytical RDT results within 15–20 minutes of testing.

Before the qPCR assessment, viral RNA extraction was performed manually using the QIAamp Viral RNA Mini Kit (QIAGEN, Hilden, Germany) for all samples. The presence of either S or E genes was considered indicative of an active SARS-CoV-2 infection. To determine the S and E genes, the samples were assessed by qPCR using the RealStar^®^ SARS-CoV-2 RT-PCR Kit 1.0 (Altona Diagnostics, Hamburg, Germany). For the reaction, 5 *µ*L of the RNA extraction was mixed with 10 *µ*L of the master mix. Internal positive and negative controls were used for quality assurance. The qPCR assessment was conducted using QuantStudio5 Biosystems (Thermo Fisher Scientific, Waltham, MA). A sample was defined as positive when the Ct value was <40 cycles. Quantitative real-time polymerase chain reaction served as the diagnostic gold standard against which the CLINITEST^®^ results were validated.

## STATISTICAL ANALYSES

The data were recorded on paper-based source documents and entered into an electronic case report form. The statistical analysis was performed using STATA 17.0 Basic Edition (StataCorp LLC, College Station, TX). The baseline characteristics of all study participants were presented using descriptive statistics. Continuous data were summarized and presented as median (interquartile range [IQR] and ranges) in the case of nonparametric data distribution. Categorical data were presented as absolute numbers and relative percentages. A *P*-value of <0.05 in statistical hypothesis testing was considered significant. The diagnostic performance of the CLINITEST^®^ was evaluated using the following quality criteria: sensitivity, specificity, positive predictive value (PPV), negative predictive value (NPV), positive and negative likelihood ratios, and the area under the receiver operating characteristic (ROC) curve.

No data were recorded between symptom onset and the participants’ presentation to the outpatient mobile test services. Therefore, the diagnostic performance of the CLINITEST^®^ was evaluated using all samples from enrolled participants, including asymptomatic, symptomatic, early, and late presenters; this group constituted the “main analysis population.” Additionally, we computed the diagnostic test performance for SARS-CoV-2-infected participants by site. Furthermore, we computed the diagnostic test performance for subpopulations of SARS-CoV-2-infected participants with increasingly higher viral loads. To do this, we restricted the population of qPCR SARS-CoV-2 positive participants into subpopulations with Ct values of <35, 30, 25, and 20, respectively.

## RESULTS

### Demographic characteristics.

From August 2021 to February 2022, 307 participants were screened and initially enrolled in this study at ISSA and CERMEL. However, 30 participants (9.8%) later withdrew consent or declined to provide two nasopharyngeal swabs, resulting in a final sample size of 277 participants and a participation rate of 90.2%. Among these, 177/277 (63.9%) were enrolled in Sindara, and 100/277 (36.1%) were enrolled in Lambaréné. Of the participants, 54.6% (150/275) were female (Table 1). The median age of the participants was 29 years (IQR: 12–55). Eighty-seven participants out of 277 (31.4%) had positive qPCR results. The median Ct value among those with a positive qPCR result was 26.5 (IQR: 23.5–29.3). In addition, 29 out of 277 (10.5%) had positive RDT results (Table 1).

**Table 1:** Baseline characteristics and average quantitative real-time polymerase chain reaction results of the study participants in Sindara and Lambaréné

Population Characteristics	Overall	Sindara	Lambaréné
Number of participants	277 (100%)	177 (63.9%)	100 (36.1%)
Sex*			
Female (*n*, %)	150 (54.6%)	100 (56.5%)	50 (51%)
Male (*n*, %)	125 (45.4%)	77 (43.5%)	48 (49%)
Age (years)			
Median (IQR) [range]	29 (12–55) [4–89]	33 (12–56) [4–89]	19 (15–32) [6–65]
Ct value^†^			
Median (IQR) [range]	26.51 (23.46–29.34) [15.5–36.2]	25.54 (22.3–25.7) [21.8–27.6]	27.07 (23.5–29.4) [15.5–36.2]

Ct = cycle threshold; IQR = interquartile range.

* Data on sex was not available for two participants.

^†^ Only includes positive quantitative real-time polymerase chain reaction results.

### Overall performance of the Siemens CLINITEST^®^ rapid COVID-19 antigen test.

The prevalence of SARS-CoV-2 infection was 31.4% (87/277; 95% CI: 26–37.2%) in the main analysis population. In the diagnostic model with SARS-CoV-2-infected participants in which the Ct value was restricted to <35 cycles, the prevalence was 30.7% (95% CI: 25.3–36.5%). In the model in which the Ct value was restricted to <30 cycles, the prevalence was 26.4% (95% CI: 21.1–32.2%). When restricted to individuals with Ct values <25 cycles, the prevalence was 13.6% (95% CI: 9.4–18.9%), and when restricted to Ct values <20 cycles, the prevalence was 3.1% (95% CI: 1.1–6.5%; Table 2).

**Table 2:** Test performance of the Siemens CLINITEST^®^ rapid COVID-19 antigen test (rapid diagnostic test) at point of care for detection of severe acute respiratory syndrome coronavirus 2 infection

Performance Characteristics in Different Populations	*N*	qPCR Positive	qPCR Negative	χ^2^ Test
Main analysis population				
RDT positive	277	29 (33.3%)*	0	<0.001
RDT negative		58 (66.7%)	190 (100%)^†^	
Restricted for qPCR SARS-CoV-2 positives with Ct value <35				
RDT positive	274	29 (34.5%)*	0	<0.001
RDT negative		55 (65.5%)	190 (100%)^†^	
Restricted for qPCR SARS-CoV-2 positives with Ct value <30				
RDT positive	258	27 (39.7%)*	0	<0.001
RDT negative		41 (60.3%)	190 (100%)^†^	
Restricted for qPCR SARS-CoV-2 positives with Ct value <25				
RDT positive	220	19 (63.3%)*	0	<0.001
RDT negative		11 (36.7%)	190 (100%)^†^	
Restricted for qPCR SARS-CoV-2 positives with Ct value <20				
RDT positive	196	5 (83.3%)*	0	<0.001
RDT negative		1 (16.7%)	190 (100%)^†^	

Ct = cycle threshold; qPCR = quantitative real-time polymerase chain reaction; RDT = rapid diagnostic test; SARS-CoV-2 = severe acute respiratory syndrome coronavirus 2. Ct cycle value not available for one positive qPCR result.

* Column percentage: sensitivity.

^†^ Column percentage: specificity.

The overall sensitivity of the CLINITEST^®^ was 33.3% (95% CI: 23.6–44.3%) in the main analysis population. In the subpopulation of SARS-CoV-2-infected participants with a Ct value restricted to <35 cycles, the sensitivity was 34.5% (95% CI: 24.5–45.7%). In the subpopulation with a Ct value restricted to <30 cycles, the sensitivity was 39.7% (95% CI: 28.0–52.3%); for individuals with a Ct value restricted to <25 cycles, the sensitivity was 63.3% (95% CI: 43.9–80.1%); and for those with a Ct value restricted to <20 cycles, the sensitivity was 83.3% (95% CI: 35.9–99.6%; Table 3).

**Table 3:** Test performance characteristics of the Siemens CLINITEST

Performance Characteristics	Main Analysis Population (including samples of all Ct values)	Restricted for qPCR SARS-CoV-2 Positives with a Ct Value below a Threshold			
		Ct Value <35	Ct Value <30	Ct Value <25	Ct Value <20
Sensitivity (95% CI)	33.3% (23.6–44.3%)	34.5% (24.5–45.7%)	39.7% (28.0–52.3%)	63.3% (43.9–80.1%)	83.3% (35.9–99.6%)
Specificity (95% CI)	100% (98.1–100%)	100% (98.1–100%)	100% (98.1–100%)	100% (98.1–100%)	100% (98.1–100%)
Positive predictive value (95% CI)	100% (88.1–100%)	100% (88.1–100%)	100% (87.2–100%)	100% (82.4–100%)	100% (47.8–100%)
Negative predictive value (95% CI)	76.6% (70.8–81.7%)	77.6% (71.8–82.6%)	82.3% (76.7–87.0%)	94.5% (90.4–97.2%)	99.5% (97.1–100.0%)
Positive likelihood ratio (95% CI)	N/A*	N/A*	N/A*	N/A*	N/A*
Negative likelihood ratio (95% CI)	0.7 (0.6–0.8)	0.7 (0.6–0.8)	0.6 (0.5–0.7)	0.4 (0.2–0.6)	0.2 (0–1.0)
Area under ROC curve (95% CI)	0.7 (0.6–0.7)	0.7 (0.6–0.7)	0.7 (0.6–0.8)	0.8 (0.7–0.9)	0.9 (0.8–1.0)
Prevalence^†^ (95% CI)	31.4% (26–37.2%)	30.7% (25.3–36.5%)	26.4% (21.1–32.2%)	13.6% (9.4–18.9%)	3.1% (1.1–6.5%)

Ct = cycle threshold; qPCR = quantitative real-time polymerase chain reaction; ROC = receiver operating characteristic; SARS-CoV-2 = severe acute respiratory syndrome coronavirus 2.

* No false positives; in this case, the “positive likelihood ratio” is infinitely high.

^†^ Prevalence of Sars-CoV-2 as determined by qPCR.

Among all samples with a negative qPCR result, all CLINITEST^®^ results were also negative. The CLINITEST^®^ demonstrated an overall specificity of 100% (190/190; 95% CI: 98.1–100%) in both the main analysis population and all diagnostic subpopulations (Table 3).

The PPV in the main analysis population was 100% (95% CI: 88.1–100%) and was also 100% in all diagnostic subpopulations (Table 3).

The NPV in the main analysis population was 76.6% (95% CI: 70.8–81.7%). In the diagnostic model involving SARS-CoV-2-infected participants with a Ct value restricted to <35 cycles, the NPV was 77.6% (95% CI: 71.8–82.6%). In the model with a Ct value restricted to <30 cycles, the NPV was 82.3% (95% CI: 76.7–87.0%). When restricted to individuals with <25 cycles, the NPV was 94.5% (95% CI: 90.4–97.2%), and when restricted to <20 cycles, the NPV was 99.5% (95% CI: 97.1–100%; Table 3).

The positive likelihood ratio could not be determined because a specificity of 100% results in a value that approximates infinity (Table 3).

The negative likelihood ratio was 0.7 (95% CI: 0.6–0.8) in the main analysis population. In the sub-population of SARS-CoV-2 infected participants with a Ct value restricted to <35 cycles, the negative likelihood ratio remained 0.7 (95% CI: 0.7–0.8). For the subpopulation with a Ct value restricted to <30 cycles, the negative likelihood ratio was 0.6 (95% CI: 0.5–0.7). When restricted to individuals with Ct values <25 cycles, the negative likelihood ratio was 0.4 (95% CI: 0.2–0.6), and when restricted to <20 cycles, the negative likelihood ratio was 0.2 (95% CI: 0–1.0; Table 3).

The area under the ROC curve was 0.7 (95% CI: 0.6–0.7) in the main analysis population. In the diagnostic model for SARS-CoV-2-infected participants with a Ct value restricted to <35 cycles, the area under the ROC curve was 0.7 (95% CI: 0.6–0.7). In the model with a Ct value restricted to <30 cycles, the area under the ROC curve was 0.7 (95% CI: 0.6–0.8). In the model with a Ct value restricted to <25 cycles, the area under the ROC curve was 0.8 (95% CI: 0.7–0.9). Finally, in the model with a Ct value restricted to <20 cycles, the area under the ROC curve was 0.9 (95% CI: 0.8–1.0; Table 3).

### Performance of the Siemens CLINITEST^®^ rapid COVID-19 antigen test by site.

The prevalence of SARS-CoV-2 infection was 4% in Sindara (7/177; 95% CI: 1.6–8%) and 80% in Lambaréné (80/100; 95% CI: 70.8–87.3%; Table 4). The sensitivity of the CLINITEST^®^ was 71.4% in Sindara (95% CI: 29–96.3%) and 30% in Lambaréné (95% CI: 20.3–41.3%), whereas the specificity was 100% in both locations (95% CI: 97.9–100% for Sindara and 95% CI: 83.2–100% for Lambaréné; Table 4). The PPV was 100% in both Sindara and Lambaréné (Table 4).

**Table 4:** Test performance characteristics of the Siemens CLINITEST per study site

Performance Characteristics	Overall Performance in Sindara	Overall Performance in Lambaréné
Sensitivity (95% CI)	71.4% (29–96.3%)	30% (20.3–41.3%)
Specificity (95% CI)	100% (97.9–100%)	100% (83.2–100%)
Positive predictive value (95% CI)	100% (47.8–100%)	100% (85.8–100%)
Negative predictive value (95% CI)	98.8% (95.9–99.9%)	26.3% (16.9–37.7%)
Positive likelihood ratio (95% CI)	N/A*	N/A*
Negative likelihood ratio (95% CI)	0.3 (0.1–0.9)	0.7 (0.6–0.8)
Area under ROC curve (95% CI)	0.9 (0.7–1.0)	0.7 (0.6–0.7)
Prevalence^†^ (95% CI)	4% (1.6–8%)	80% (70.8–87.3%)

ROC = receiver operating characteristic.

* No false positives; in this case, the “positive likelihood ratio” is infinitely high.

^†^ Prevalence of Sars-CoV-2 as determined by qPCR.

The NPV in Sindara was 98.8% (95% CI: 95.9–99.9%), whereas in Lambaréné, it was 26.3% (95% CI: 16.9–37.7%; Table 4). The positive likelihood ratio could not be determined because a specificity of 100% results in a value that approximates infinity (Table 4). The negative likelihood ratio in Sindara was 0.3 (95% CI: 0.1–0.9), and in Lambaréné, it was 0.7 (95% CI: 0.6–0.8). The area under the ROC curve in Sindara was 0.9 (95% CI: 0.7–1), and in Lambaréné, it was 0.7 (95% CI: 0.6–0.7). Among those with a positive qPCR result, five out of seven had a positive RDT result in Sindara (71.4%), whereas, in Lambaréné, 24 out of 56 (70%) had a positive RDT result (Table 5).

**Table 5:** Test performance characteristics of the Siemens CLINITEST per study site

Performance Characteristics per Study Site	*N*	qPCR Positive	qPCR Negative	χ^2^ Test
Sindara				
RDT positive	177	5 (71.4%)	0 (0%)	<0.000
RDT negative		2 (28.6%)	170 (100%)	
Lambaréné				
RDT positive	100	24 (30%)	0 (0%)	<0.005
RDT negative		56 (70%)	20 (100%)	

qPCR = quantitative real-time polymerase chain reaction; RDT = rapid diagnostic test.

## DISCUSSION

In this study, we evaluated the diagnostic performance of the CLINITEST^®^ at two sites in rural Gabon. Our results indicated an overall test sensitivity of 33.3% (95% CI: 23.6–44.3%), which increased to 83.3% (95% CI: 35.9–99.6%) when restricted to a Ct value <20. The overall test specificity across all analyzed populations was 100% (190/190; 95% CI: 98.1–100%).

Point-of-care testing is a strategy to address diagnostic challenges in resource-limited settings.^8^ Rapid diagnostic tests with high sensitivity and specificity are crucial for an effective control strategy to reduce the spread of SARS-CoV-2 infection. High sensitivity ensures that all truly infected individuals will be detected, whereas high specificity ensures that genuinely noninfected individuals are correctly classified and, consequently, not unnecessarily quarantined. Much research has evaluated the diagnostic performance of different SARS-CoV-2 antigen RDTs.^8^^,^^10^^,^^11^^,^^14^^,^^16^^,^^20^^,^^21^ In 2021, SIEMENS Healthineers validated the CLINITEST^®^ using a total of 865 samples, comprising 120 qPCR-positive and 745 qPCR-negative samples stemming from seven different sites in the United States. Based on this assessment, the CLINITEST^®^ demonstrated a sensitivity of 98.3% (95% CI: 94.1–99.8%) and a specificity of 99.6% (95% CI: 98.8–99.9%).^12^ Here, we report the diagnostic performance of the CLINITEST^®^ in Sindara and Lambaréné, Gabon, using 277 samples. Our results demonstrated that the CLINITEST^®^ had low overall sensitivity compared with qPCR but excellent specificity. We observed increased sensitivity of the CLINITEST^®^ for incrementally lower Ct value ranges, reaching a peak sensitivity of 83.3% (95% CI: 35.9–99.6%) in the “Ct <20” subpopulation. This indicates a strong negative correlation between the Ct value of the qPCR and RDT sensitivity, suggesting better performance in participants with a high viral load. This is supported by values for model fit, whereby the “Ct <20” sub-population model had the most favorable value (area under the ROC curve of 0.9). This finding is comparable with other studies evaluating the CLINITEST^®11,13,22–24^ and is plausible because high viral loads are more easily detectable than lower ones. Navero-Castillejos et al. conducted a prospective study from November 2020 to January 2021 to evaluate the test performance of the CLINITEST^®^, among other RDTs, using a sample of 130 participants. They reported that the CLINITEST^®^ had a sensitivity of 79.4%, which increased when the Ct value decreased.^23^

Olearo et al.^10^ evaluated the analytical performance of the CLINITEST^®^ in Hamburg, Germany, and reported a much lower sensitivity of 54.9% (95% CI: 43.4–65.9%). Most studies evaluating the performance of various antigen RDTs (Ag-RDTs) were conducted in Western countries in Europe and America, with only a few studies conducted in Africa and Asia. In summary, the performance studies of these Ag-RDTs conducted in Europe showed a higher overall sensitivity compared with those conducted in Africa and Asia, suggesting impaired test performance of Ag-RDTs in those settings. Potential reasons for this impaired performance in Asia and Africa may include freeze and thaw procedures during shipment.^25^ These freeze and thaw procedures may have contributed to the low sensitivity in our study because the CLINITEST^®^ was shipped from Germany to Gabon.

Furthermore, differences in ambient temperatures between Europe and Africa might have contributed to variations in sensitivity. Haage et al. assessed the diagnostic sensitivity and specificity of several commercially available RDTs for SARS-CoV-2 antigen using different storage and operational temperatures (2–37°C). They concluded that the sensitivity of five out of 11 SARS-CoV-2 Ag-RDTs was reduced 10-fold when testing was performed at 37°C compared with temperatures between 15°C and 30°C. Moreover, they reported that the sensitivity of eight out of 11 SARS-CoV-2 Ag-RDTs was reduced more than 10-fold when the tests were stored at 37°C for 3 weeks.^17^

High sensitivity is crucial for effective case management and reducing the spread of infection with SARS-CoV-2. Rapid diagnostic tests can improve testing strategies and enhance accessibility in resource-limited countries.^26^ However, if the sensitivity is very low, a negative test result does not rule out an infection with SARS-CoV-2. As indicated by our study, the CLINITEST^®^ does not detect all infected participants. Therefore, the overall test performance of CLINITEST^®^ does not fulfill the quality criteria set by the WHO, which requires a sensitivity of at least 80% for approval.^26^ Nonetheless, the sensitivity of CLINITEST^®^ was above 80% in a sub-population of SARS-CoV-2 infected participants with a high viral load (Ct value of <20). This finding has three potential implications for the utility of the CLINITEST^®^ in detecting SARS-CoV-2-infected participants in sub-Saharan African countries. First, the CLINITEST^®^ is likely effective in identifying highly contagious participants because contagiousness has been reported to be positively correlated with Ct.^27^ Second, some authors have suggested that high viral loads are present during the early stages of infection compared with later stages.^28^ Third, high viral loads have been reported in participants with severe symptoms, although it remains inconclusive whether this phenomenon is restricted to older participants with SARS-CoV-2 infection.^29^ Thus, the favorable performance of CLINITEST^®^, as indicated in the “Ct <20” subpopulation model, likely extends to highly contagious SARS-CoV-2-infected participants, early presenters with SARS-CoV-2 infection, and potentially, those with severe COVID-19 symptoms. Therefore, the CLINITEST^®^ may still be valuable, particularly in settings with limited access to qPCR testing. Furthermore, repeated daily testing could increase sensitivity for symptomatic participants with initially negative results but characteristic symptoms. However, it is uncertain whether such a strategy would truly enhance diagnostic sensitivity, and it therefore requires previous evaluation.^26^

High specificity is crucial for accurately classifying noninfected participants as noninfected. A highly specific RDT contributes to effective case management and ensures that quarantining measures are imposed only on those infected with SARS-CoV-2. In our study, the specificity was 100% (95% CI: 98.1–100%) overall and in all other diagnostic models, which exceeds the current specificity threshold (i.e., ≥97%) recommended by the WHO for COVID-19 RDTs.^26^ These consistently high specificity values indicate that false positive results are unlikely.

According to the WHO’s recommendations, NPV and PPV should be considered if an ongoing transmission rate is defined as a test positivity rate of ≥5%.^30^ From March 2020 to December 2021, the prevalence of SARS-CoV-2 infection in Gabon was 1.6% (37,342 SARS-CoV-2 cases out of 2,341,000 inhabitants), indicating a very low transmission rate. With a specificity of 100%, the PPV in our study overall and across all diagnostic models was 100%, making it highly favorable for case management; these predictive values are based on a qPCR-determined SARS-CoV-2 prevalence ranging from 31.4% to as low as 3.1%. However, the WHO recommends that NAAT, such as qPCR, be performed in low transmission settings like Gabon.^30^

The prevalence of SARS-CoV-2 infection in Sindara was 4% (7/177, 95% CI: 1.6–8%), and in Lambaréné, it was 80% (80/100; 95% CI: 70.8–87.3%; Table 4). However, rather than assuming that there are truly such vastly different rates of SARS-CoV-2 infection among the wider populations of Lambaréné and Sindara, an alternative explanation is suggested for this phenomenon. The Institut de Santé de Sindara is a relatively novel satellite site of CERMEL, founded in 2021, compared with CERMEL, which was founded in 1981.^18^ Therefore, it is believed that the novelty of ISSA itself may have attracted the inhabitants of Sindara, prompting many people to visit the research center and participate in the study out of mere curiosity, regardless of whether they were asymptomatic or symptomatic. Subsequently, given that many noninfected individuals participated in Sindara, the pretest probability of SARS-CoV-2 infection and its prevalence are lower. In contrast, inhabitants of Lambaréné have been familiar with CERMEL for decades; therefore, it is believed that participants mainly consulted the diagnostic services of CERMEL if they were symptomatic and sought medical care there.^18^ Consequently, among such a group, the pretest probability for SARS-CoV-2 infection is expected to be much higher than in Sindara. However, even if the prevalences of SARS-CoV-2 infection may have differed between Sindara and Lambaréné, this has not affected the diagnostic performance characteristics of the CLINITEST^®^ because the sensitivity, specificity, positive likelihood ratio, negative likelihood ratio, and area under the ROC curve are parameters that are independent of prevalence.

The sensitivity of the CLINITEST^®^ was 71.4% (95% CI: 29–96.3%) in Sindara and 30% (95% CI: 20.3–41.3%) in Lambaréné (Table 4). The difference in sensitivity between Sindara and Lambaréné most likely results from different characteristics of SARS-CoV-2 infection in the two settings. The median Ct value in Sindara was higher (25.5 [IQR: 22.27–25.7]), making it easier for the CLINITEST^®^ to detect SARS-CoV-2 compared with Lambaréné, where the median Ct value was 27.07 (IQR: 23.5–29.5; Table 1). The different characteristics of SARS-CoV-2 infection in Sindara and Lambaréné did not affect the specificity of the CLINITEST^®^. The PPV did not differ significantly between Sindara and Lambaréné. However, there was a substantial difference in the NPV between Sindara and Lambaréné. Whereas the NPV was 98.8% (95% CI: 95.9–99.9%) in Sindara, it was 26.3% (95% CI: 16.9–37.7%) in Lambaréné, which can be explained by differences in prevalence because predictive values are dependent on prevalence. This shows that the CLINITEST^®^ may be a valuable tool for clinical testing in a broader target population, including both asymptomatic and symptomatic individuals with a relatively low pretest probability for SARS-CoV-2 infection, given that both PPV and NPV were favorable in our low-prevalence setting (i.e., Sindara). However, the CLINITEST^®^ is likely not an appropriate tool for definitive diagnosis in a central SARS-CoV-2 testing laboratory, in which the pretest probability of SARS-CoV-2 infection is expected to be high. At the same time, the CLINITEST^®^ may still be a helpful tool for correctly identifying SARS-CoV-2-infected individuals. Because of its high PPV, the CLINITEST^®^ is expected not to be useful for ruling out SARS-CoV-2 infection, given its low NPV in high prevalence settings.

Our study had some limitations. First, no a priori sample size calculation was performed; between August 2021 and February 2022, we invited all participants presenting for diagnostic SARS-CoV-2 infection services to participate in the study. Second, we did not assess the symptoms associated with SARS-CoV-2 infection or the severity of infected and diseased participants. However, we presented the diagnostic performance characteristics for different Ct value ranges that serve as surrogates for performance in symptomatic (i.e., those with low Ct values) and asymptomatic participants (i.e., those with high Ct values). Third, samples were collected from rural regions in Sindara, where, at the time of sample collection, resources maintaining the cold chain, electricity, and ambient temperature were limited. In addition, the diagnostic turnaround times were longer in Sindara than in Lambaréné, where samples were shipped directly to CERMEL. Fourth, the CLINITEST^®^ was performed in Sindara at the POC; swabs were sent to CERMEL for qPCR confirmation. Thus, the sample quality may not be identical, potentially impacting test performance. Fifth, we did not determine antigen levels in samples as suggested by Khalid et al.^25^ Despite these limitations, it may be possible to improve the test performance of the CLINITEST^®^ for African settings when regional conditions are considered. Moreover, qPCR test performance was high regardless of the sampling method used.^25^

## CONCLUSION

In conclusion, our study evaluated the test performance of the CLINITEST^®^. The CLINITEST^®^ had an overall sensitivity of 33.3% (95% CI: 23.6–44.3%) and a specificity of 100% (95% CI: 98.1–100%). Therefore, the CLINITEST^®^ is not a viable alternative to qPCR for effective monitoring, contact tracing, or outbreak control. However, our statistical models suggest that the test may be valuable in detecting SARS-CoV-2-infected participants with a high viral load, including highly contagious individuals, early presenters, and potentially, those with COVID-19 and severe symptoms. Future studies on Ag-RDTs for SARS-CoV-2 should be conducted in tropical settings to evaluate their performance in real-world situations. The development of new RDTs, which should not only be appropriate for the European countries where most Ag-RDTs have been developed, should also include the SSA setting.

## Data Availability

The datasets generated and analyzed for this study cannot be shared because the data are still being used for further manuscripts. Once all analyses are complete, the data will be available upon request.
